# Antifungal Activity of Lipopeptides From *Bacillus* XT1 CECT 8661 Against *Botrytis cinerea*

**DOI:** 10.3389/fmicb.2018.01315

**Published:** 2018-06-26

**Authors:** Laura Toral, Miguel Rodríguez, Victoria Béjar, Inmaculada Sampedro

**Affiliations:** ^1^Xtrem Biotech S.L., European Business Innovation Center, Granada, Spain; ^2^Department of Microbiology, Faculty of Pharmacy, University of Granada, Granada, Spain; ^3^Biomedical Research Center (CIBM), Biotechnology Institute, Granada, Spain

**Keywords:** *Bacillus* XT1, lipopeptides, antifungal activity, *Botrytis cinerea*, antioxidant activity

## Abstract

This work aims to explore the capacity of a *Bacillus methylotrophicus* (later heterotypic synonym of *Bacillus velezensis*) strain named XT1 CECT 8661 against the necrotrophic plant pathogen *Botrytis cinerea* and to identify the compounds responsible for its activity. Q_TOF electrospray mass spectrometry analysis allows us to detect several lipopeptides – surfactin, bacillomycin, and fengycin – in XT1 cultures. *In vitro* antibiosis studies demonstrated the efficiency of the lipopeptide fraction for the inhibition of fungal growth. In fact, microscopy studies (SEM/TEM) revealed, an alteration of the morphology of the phytopathogen in interaction with lipopeptides, with resistance structures appearing in the early stages of growth of the fungus. Our studies, carried out with tomatoes, grapes, and strawberries have demonstrated the efficiency of *Bacillus* XT1 CECT 8661 lipopeptides against *B. cinerea* infection and it capability to trigger the antioxidant activity in fruit. Overall, the results of this study highlight the potential of lipopeptides of this strain as an effective biological control agent against the colonisation of *B. cinerea*.

## Introduction

*Botrytis cinerea* [teleomorph: *Botryotinia fuckeliana* (de Bary) Whetzel] is a necrotrophic fungi known to be the cause of grey mould. These fungi can infect more than 200 plant species, including horticulturally important crops (Gao et al., 2018). Therefore, it has significant economic relevance, causing huge economic losses (Dean et al., 2012). The ability to produce conidiophores that contain conidia gives it the ability to remain quiescent until conditions are favourable enough to produce the infection (Gao et al., 2018). The production of lytic enzymes along with other phytotoxic metabolites induces cell death in plant tissues, mainly affecting those in a state of senescence or with wounds on their surface (Finiti et al., 2014; Gonzalez-Fernandez et al., 2015; Yu et al., 2015; Yahaya et al., 2016). The aforementioned ubiquity, together with the capacity to produce resistant structures and the high mutation rate of *B. cinerea*, makes the fight against this fungus a challenging task (Gonzalez-Fernandez et al., 2015; Haidar et al., 2016).

Currently, the most popular treatment to combat grey mould is the extensive use of pesticides; however, recent regulations of these products have considerably restricted the possibility of their use. These pesticides produce residual waste and contaminate ground water increasing the risks for human health and the environment (Perez-Garcia et al., 2011; Romanazzi et al., 2012; Finiti et al., 2014). As a consequence, one of the biggest challenges for sustainable agriculture is the development of environmentally friendly alternatives such as the use of microorganisms. The biological control through bacterial strains has been an objective of particular interest due to their multiple modes of action against different plant diseases. One of these mechanisms is the production of a wide variety of biologically active compounds with great potential for biotechnological applications (Gond et al., 2015; Martinez-Hidalgo et al., 2015; Mnif et al., 2016).

Although several microorganisms have been described as potential candidates for biological control agents, numerous research studies have focused on members of the genus *Bacillus*. Species from this genus have been considered biologically safe and are commonly used in agriculture. The sporulation capacity of these microorganisms gives them a high resistance, high ubiquity in diverse habitats and stability in formulated products (Ongena and Jacques, 2008; Tanaka et al., 2015; Mnif et al., 2016). Members of the genus *Bacillus* are well known for their capacity to colonise roots, promote plant growth (PGPR) and induce systemic resistance mechanism in plants (Sicuia et al., 2015). In addition, they can produce a broad spectrum of biologically active molecules, with potential antimicrobial and antifungal properties. One of the major factors related with the antifungal activity of members of the genus *Bacillus* is due to the production of lipopeptides (Ongena and Jacques, 2008).

Lipopeptides are low-molecular-weight cyclic amphiphilic oligopeptides synthesised by multi-enzyme complexes called non-ribosomal peptide synthetases (NRPSs) (Romero et al., 2007; Gond et al., 2015; Han et al., 2015; Deng et al., 2017). Species from the genus *Bacillus* produce molecules which are mainly classified into three families depending on their amino-acid sequence: surfactin, iturin, and fengycin. These families share a cyclic β-amino or β-hydroxy fatty acid linked to a lipid tail (Tapi et al., 2010; Sumi et al., 2014; Jemil et al., 2017). Biological activities may differ from one compound to another depending on the type of amino-acid residues, the cyclisation of the peptide and the length and branching of the fatty acid chain (Ongena and Jacques, 2008; Frikha-Gargouri et al., 2017).

Different studies have demonstrated the activity of lipopeptides produced by *Bacillus subtilis* (Farace et al., 2015; Wang et al., 2015; Arroyave-Toro et al., 2017). However, the information about the antifungal activity of lipopeptides produced by *B. methylotrophicus* against *B. cinerea* is almost non-existent.

The objective of this study is to analyse the antifungal activity of *B. methylotrophicus* XT1 CECT 8661 (later reclassified as heterotypic synonym *B. velezensis*) (Ruiz-García et al., 2005) against *B. cinerea*. For this purpose, the lipopeptides produced by *B. methylothrophicus* by this strain were identified, the genes involved in their biosynthesis were detected and their antifungal activity was tested *in vitro*. Alterations of the morphology of the phytopathogen in interaction with these macromolecules was examined via microscopy studies. Furthermore, studies of antibiosis *in vivo* and determination of antioxidant compounds on grapes, tomatoes, and strawberries were carried out in order to demonstrate the ability of these compounds to protect against *B. cinerea* infection and to activate antioxidant mechanisms. To the best of our knowledge, this is the first report that describes the ability of lipopeptides to trigger the antioxidant activity in fruit, a mechanism involved in the elicitation of an induced systemic resistance phenomenon.

## Materials and Methods

### Bacterial and Fungal Strains

The bacterial strain used in this study was the patented strain *Bacillus* XT1 CECT 8661, licenced to Xtrem Biotech S.L., which was isolated from a rhizospheric soil sample in the south of Spain (Béjar et al., 2014). The strain was originally classified as *B. methylotrophicus* and it was later reclassified as a heterotypic synonym of *B. velezensis*. It was routinely cultivated on a nutrient broth and nutrient agar at 28°C. The phytopathogenic fungus *B. cinerea* was kindly provided by the University of Zaragoza (Spain) and was maintained on potato dextrose agar (PDA) and potato dextrose broth (PDB) and incubated at 24°C.

### Antifungal Activity of XT1 Strain

The antifungal activity of XT1 strain against *B. cinerea* was determined in both solid and liquid mediums. For the solid assay strain XT1 was spread on a 1 cm^2^ area on one side of a PDA agar plate (at 1 cm from the plate wall) and an 8-mm-agar disc of the mycelium of fungi was deposited on the opposite side. The maximum and minimum values of the fungal mycelium radius obtained were measured after a 15-day incubation period at 25°C. The results were expressed as a percentage of the mycelium inhibition rate (IR% = A - B / A × 100) where A was the maximum value of the mycelium radius and B was the minimum value. For the antifungal assay in liquid medium, first, the time in which the antifungal activity was the maximum was determinate cultivating the strain XT1 in MOLP (medium optimal for lipopeptide production) (Ahimou et al., 2000) at different times (24, 48, 72, and 96 h), then the supernatant was tested against *B. cinerea* following the procedure described below. A 15-day culture of *B. cinerea* in PDB was crushed in a breaker and filtered with gauze, all under sterile conditions. The spore concentration was adjusted to 5 × 10^7^ conidia mL^-1^ and penicillin G (2.5 mg mL^-1^) and streptomicin (10 mg mL^-1^) was added to the spore suspension. The experiment was carried out on multiwell culture plates (Cellstar^∗^) with 48 wells where 900 μl of the spore solution was subjected to 300 μL of different times XT1 supernatant, obtained after centrifugation of the XT1 culture in MOLP at 10000 rpm 20 min. Inoculated medium with cycloheximide 50 μg mL^-1^ was used as a positive control for growth inhibition, whilst PDB inoculated with spore suspension without treatment was considered as the negative control. The plates were incubated at 25°C for 7 days. The results were obtained by observing the presence or absence of fungal growth (Frikha-Gargouri et al., 2017).

### Lipopeptide Production

Three different liquid media have been tested for lipopeptide production: MOLP medium (Ahimou et al., 2000); SG medium (Schaeffer et al., 1965; Leighton and Doi, 1971), and a commercial concentrated beef medium (CM) (ox concentrate 43% and yeast extract 24%). Lipopeptides were extracted according to Yazgan et al. (2001) with slight modifications. Briefly, the culture supernatant of strain XT1 was subjected to an organic extraction with one volume *n*-butanol three times using a decantation funnel. Then, the organic phase was evaporated with a vacuum concentrator.

### Antifungal Activity of the Lipopeptides Produced by XT1

Lipopeptide antifungal activity was tested by preparing 20 mL of lipopeptide solution at different concentrations: 20, 10, 8, 6, 4, 2, 1, and 0.5 mg mL^-1^ (w/v in distilled water) in 50 mL tubes. Then 0.8 g PDA was added to each lipopeptide solution; the negative control was made with untreated PDA medium. The tubes were then sterilised and the medium were poured into 90 mm Petri plates. Next, a fungal plug of 15 days of mycelial growing *B. cinerea* was deposited in the middle of each plate and maintained at 25°C for 15 days. After that, the mycelial growth inhibition percentage was calculated after 15 day’s incubation by the comparison between the diameter of mycelial growing in the control plates and the treatments according to the following formula: mycelial growth inhibition = 100-[(diameter of control mycelium diameter of mycelium in lipopeptide medium / diameter of control mycelium) × 100] (Borah et al., 2016).

### Determination of Minimal Inhibitory Concentration (MIC) and Minimal Fungicidal Concentration (MFC) of the Lipopeptides

The minimal inhibitory concentration (MIC), defined as the smallest concentration of lipopetides that inhibits the fungal growth totally and the minimal fungicidal concentration (MFC), defined as the lowest concentration of lipopeptides capable of killing the fungi, was determined. The experiment was carried out in liquid medium according to the protocol described previously in the “*Antifungal Activity of XT1 Strain*” section. However, in this case 900 μl of the spore solution was exposed to 300 μL of lipopeptide dilutions 20, 10, 8, 6, 4, 2, 1, and 0.5 mg mL^-1^. The plates were incubated at 25°C for 7 days. The results were obtained by observing the presence or absence of fungal growth. The whole content from each well, where there was no growth of *B. cinerea*, was passed to tubes with PDB medium and incubated at 25°C for 7 days for the determination of MFC (Frikha-Gargouri et al., 2017).

### Stability of Lipopeptides to Heat and pH

The antifungal activity of a lipopeptide solution (10 mg mL^-1^) from XT1 was determined after 10, 30, and 60 min of heating at 100°C and after 20 min at 121°C. The stability was also determined at different pH in the range comprised between 3 to 12 (Ghribi et al., 2012). The antifungal activity was tested in liquid medium according to the method described above (Frikha-Gargouri et al., 2017).

### Genetic Characterization of Lipopeptides

Genes encoding NRPS production were amplified by polymerase reaction chain (PCR) from genomic DNA of the XT1 strain. PCR was carried out using the specific and degenerated primers described in **Table 1**. PCR amplifications were achieved in 50 μL mixtures with PCR buffer, 2 mM MgCl_2_, 4 mM of each primer, 5U Taq polymerase, 0.2 mM of each dNTP, and 80–100 ng of genomic DNA. The amplification conditions were: 95°C for 5 min, 40 cycles of 94°C for 1 min, annealing temperature for 1 min, 72°C extension for 1 min; and a final extension at 72°C for 10 min. The annealing temperatures were 45, 43, 50, 53, and 50°C for, Af2/Tf1, As1/Ts2, BmyBF/BmyBR, ItuDF/ItuDR, and SrfA3/LicA3 primers, respectively. The amplification products were analysed by electrophoresis in a 2% (w/v) agarose gel.

**Table 1:** PCR primers of lipopeptides biosynthesis genes in *Bacillus* XT1.

Lipopeptide	Gene	Primers	Primer sequences (5′→3′)	PCR product size (bp)	Reference
Surfactin/Lichenicin	*srf*A3*/lic*A3	SrfA3/licA3(F)	CAAAAKCGCAKCATACCAAKTTGAG	268	Randall Simpson et al., 2011
		*Srf*A3*/lic*A3 (R)	AGCGGCAYATATTGATGCGGYTC		
Fengycin	*fen*C	Af2 (F)	GAATAYMTCGGMCGTMTKGA	443–455	Tapi et al., 2010
		Tf1 (R)	GCTTTWADKGAATSBCCGCC		
Surfactin	*srf*A-A	As1 (F)	CGCGGMTACCGVATYGAGC	419–431	Tapi et al., 2010
		Ts2 (R)	ATBCCTTTBTWDGAATGTCCGCC		
Bacillomycin	*bmy*B	BmyB (F)	GAATCCCGTTGTTCTCCAAA	370	Mora et al., 2011
		BmyB (R)	GCGGGTATTGAATGCTTGTT		
Iturin	*itu*D	ItuD (F)	TTGAAYGTCAGYGCSCCTTT	482	Chung et al., 2008
		ItuD (R)	TGCGMAAATAATGGSGTCGT		


### Identification of Lipopeptides Using UPLC – HDMS Q-TOF

The residue obtained from lipopeptide extraccion was dissolved in 10% methanol and analysed by high-pressure liquid chromatography (UPLC) (Acquity UPLC^®^ BEH300, Waters) coupled to a high definition mass spectrometry (SYNAPT G2 HDMS Q-TOF. Waters). Mass spectrometry was carried out by positive ionisation electrospray (ESI+). The obtained data were processed by the MassLynx^TM^ software (Waters).

### Scanning Electron Microscopy and Transmission Electron Microscope of Mycelia Treated With Lipopeptides

Solid culture medium plates with the negative control treatments and lipopeptides 10 mg⋅mL^-1^ from the previously experiment described in the “*Antifungal Activity of the Lipopeptides Produced by XT1*” section, were used to observe the morphology of *B. cinerea*. The samples were fixed and observed in a FIB-FESEM (CrossBeam NVision 40^®^, Carl Zeiss SMT) Scanning Electron Microscope and Transmission Electron Microscope.

### Bioassay Against *B. cinerea* in Grapes, Strawberries, and Tomatoes

Antifungal activity of lipopeptides produced by XT1 was tested on grapes, strawberries, and tomatoes. *B. cinerea* was grown for 15 days on a PDA medium at 25°C and conidia were collected with sterile distilled water and filtered through four layers of sterile cheesecloth. The surface of the fruit was sterilised with 5% NaOCl for 5 min and rinsed three times with plenty of sterile water. Wounds of 3 mm were performed with a sterile scalpel on the surface of the grapes, then 15 μl of a 20 mg⋅mL^-1^ solutions of lipopeptides were applied on the injury. One hour later, when the fruit was dried at room temperature, 15 μL of a suspension of *B. cinerea* 10^8^ conidia mL^-1^ were inoculated into the wound. Strawberries and tomatoes were cut into slices and treated by spray with the same lipopeptide solution as in the previous case. Then, after 1 h, the fruit was infected by spraying with the conidia suspension of *B. cinerea.* All the treatments were incubated at 25°C, 70% humidity for 6 days. For each treatment, a total of nine fruits were used and three technical replicates were performed. Effect was measured and expressed as disease incidence (% of infected fruit).

### Antioxidant Activity on Fruit Treated With Lipopeptides

The antioxidant activity was measured extracting the soluble phenols and by FRAP assay (ferric iron reducing antioxidant power assay). Total soluble phenols were extracted from 0.1 g of lyophilized fruits with 10 mL of 80% methanol and 0.1% hydrochloric acid. The mixture was placed in the dark at 4°C for 2 h. The supernatant was filtered and the extract was used for the determination of the phenol content and the FRAP assay. The amount of total phenols was determined according to the Folin-Ciocalteu’s procedure described by Ribereau-Gayon (1968) with slight modifications. The phenol content was estimated from a standard curve of gallic acid (GAE) and the results expressed as mg of gallic acid 100 g^-1^ d.w (dry weight). In FRAP assay an extract of fruit (0.2 mL) (prepared as for phenol determination) was added to 2 mL of FRAP solution [0.25 mol L^-1^ acetate buffer (pH 3.6) containing 1 mmol L^-1^ 2,4,6-tris(2-pyridyl)-s-triazine (TPTZ) and 20 mmol L^-1^ FeCl_3_.6H_2_O] and incubated 5 min at room temperature measuring the absorbance at 593 nm. A standard of 1 mmol L^-1^ L-ascorbic acid in distilled water was prepared. Results were expressed as mmol L^-1^ of Fe^2+^ equivalents 100 g^-1^ dry weight (Benzie and Strain, 1999).

### Statistical Analyses

Data obtained were subjected to ANOVA and multiple pair-wise comparisons were performed by the Duncan’s multiple range test.

## Results

### Antifungal Activity of *Bacillus* XT1

Antifungal production in the supernatant was detected after 24 h of aerobic culture and reached its maximum at 72 h. The XT1 strain showed antifungal activity against *B. cinerea* in both solid and liquid media (inhibition rates of 60 and 100%, respectively).

### Lipopeptide Production

The production of lipopeptides was tested in different culture media. The data showed that MOLP medium increased the lipopeptide production compared with other media. The best production yield was obtained with this medium (10 g L^-1^); however, the production with other media such as SG and the commercial medium (CM) decreased the production of lipopeptide to 2.8 and 2.13 g L^-1^, respectively.

### Antifungal Activity of the Lipopeptides Produced by *Bacillus XT1*

**FIGURE 1:**
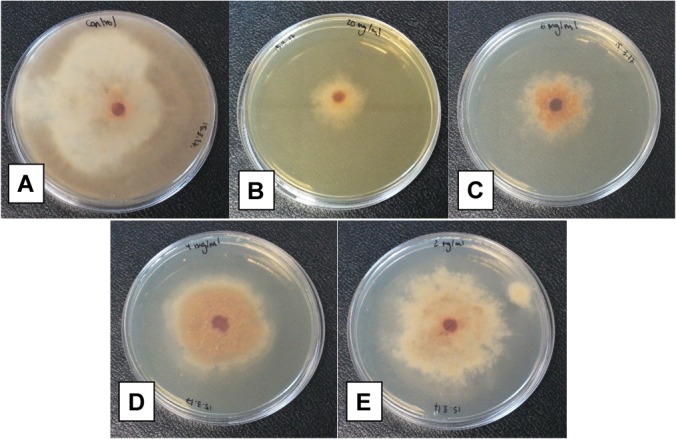
Antifungal activity of *Bacillus* XT1 lipopeptides toward *Botrytis cinerea*. Effect of different concentrations on the antifungal potency: negative control **(A)** and in the presence of: 10 mg mL^-1^ lipopeptide **(B)**, 6 mg mL^-1^ lipopeptide **(C)**, 4 mg mL^-1^ lipopeptide **(D)**, and 2 mg mL^-1^ lipopeptide **(E)**.

The antifungal activity of *Bacillus* XT1 lipopeptides toward *B. cinerea* was also analysed. The results demonstrated that lipopeptides produced by XT1 inhibit the growth of *B. cinerea*. Inhibition rates of 72, 48, 30, and 19% of the mycelium diameter were observed for the concentrations of lipopeptides of 10, 6, 4, and 2 mg mL^-1^, respectively, after 15 days of treatment (**Figure 1**). In general, lipopeptides from XT1 showed antagonistic activity against *B. cinerea* across a broad spectrum of concentrations in a dose response manner (**Figure 1**).

### Determination of Minimal Inhibitory Concentration (MIC) and Minimal Fungicidal Concentration (MFC) of the Lipopeptides

**FIGURE 2:**
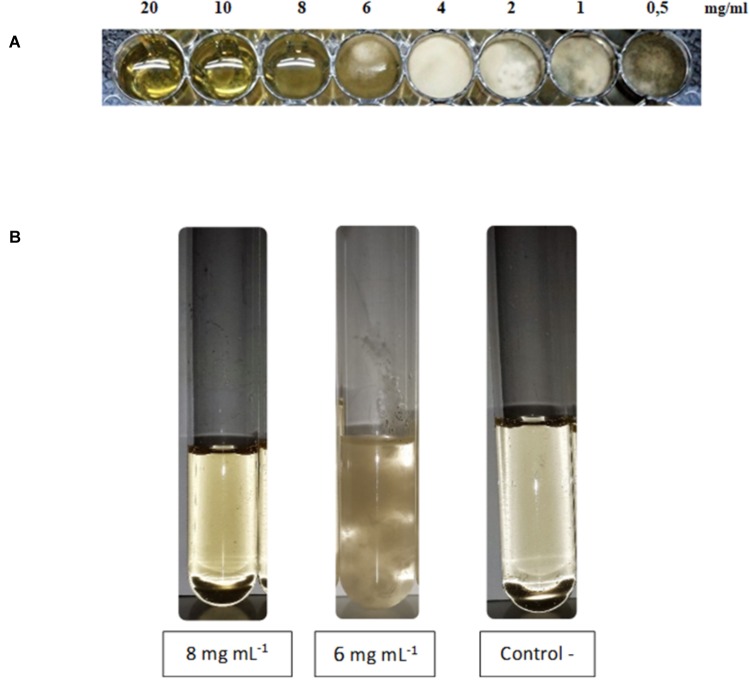
Minimal inhibitory concentration (MIC) **(A)** and minimal fungicidal concentration (MFC) **(B)** of lipopeptides from XT1.

Lipopeptides from XT1 were also tested in multiwell culture plates with 48 wells to test the MIC and in culture tubes to test the MFC. As shown in **Figure 2A** lipopeptides concentrations tested ranging from 20 to 0.5 mg mL^-1^ and a significant inhibitory effect of lipopeptides was observed at concentrations as low as 8 mg mL^-1^ which corresponds to the MIC. The MFC was also 8 mg mL^-1^ (**Figure 2B**).

### Stability of Lipopeptides to Heat and pH

The antifungal potency was not affected by any heat treatment. However, the antifungal potency of lipopeptides from XT1 was affected at pH 3 and pH 12. The optimum range of pH for the maximum antifungal efficacy was between 7 and 9.

### Detection of Genes Involved in Lipopeptide Biosynthesis

Genomic analysis of *Bacillus* XT1 indicates that it contains gene clusters for non-ribosomal lipopeptide synthetases. Amplicons of the expected sizes were obtained for fengycin, surfactin, bacillomycin, and iturin genes *srf*A-C, *fen*C, *srf*A-A, *bmy*B, and *itu*D.

### Identification of Lipopeptides Using UPLC – HDMS Q-TOF

**FIGURE 3:**
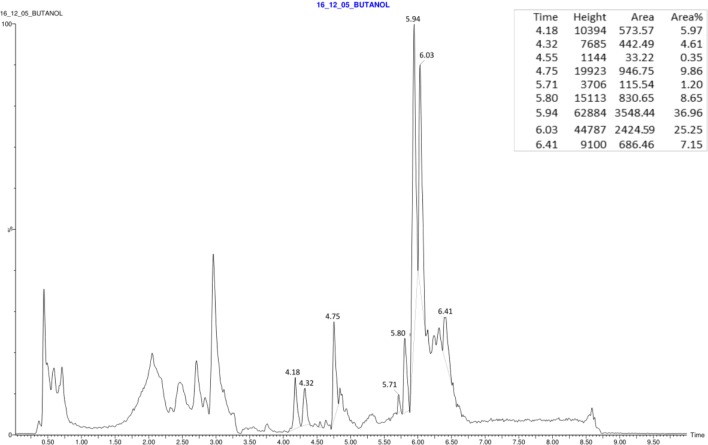
Total ion chromatogram (TIC) spectrum obtained from the surfactant of *Bacillus* XT1.

Electrospray quadrupole time-of-flight mass spectrometry (Q-TOF MS) analyses were carried out in this study to identify the metabolites produced by XT1. **Figure 3** illustrates the total ion chromatogram (TIC) spectrum of the lipopeptide extract from a XT1 culture supernatant.

These analyses showed that the strain XT1 produces several forms of different lipopeptides. Four known surfactins with an acyl chain ranging from C12 to C15 were detected, whereas three known bacillomycins D (C14, C15, and C16) were also detected. Two peaks corresponding to the fengycin A and fengycin B were also observed (**Table 2**). There were no mass signals for iturin.

**Table 2:** Lipopeptide production by *Bacillus* XT1 as detected by Q-TOF MS.

Lipopeptide	Fatty chain length	[M+H]^+^	Retention time (min)	Area (%)	Peak intensity
Bacillomycin D	C14	1031.5431	4.18	5.97	1.02e4
	C15	1045.5585	4.32	4.61	8.35e3
	C16	1059.5726	4.55	0.35	1.31e3
Fengycin A	C16	1463.8038	4.75	9.86	4.81e3
Fengycin B	C15	1477.8329	4.86	0.60	1.65e3
	C16	1491.8481	4.86	0.60	1.01e3
Surfactin	C12	994.6413	5.71	1.20	4.22e3
	C13	1008.6564	5.80	8.65	1.06e4
	C14	1022.6729	5.94	36.96	8.40e4
	C15	1036.6867	6.03	25.25	6.60e4


**FIGURE 4:**
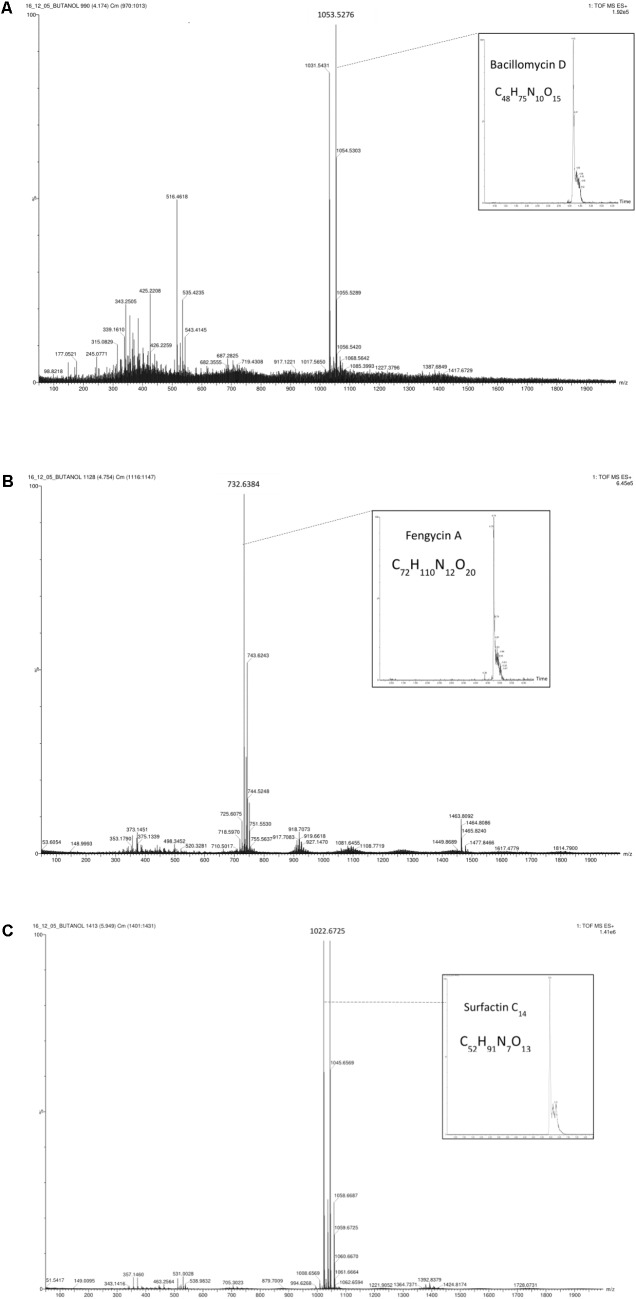
Quadrupole time-of-flight mass spectrometry (Q-TOF MS) spectra obtained from the surfactant produced by *Bacillus* XT1: protonated linear derivatives of the [M+H] of bacillomycin D **(A)**, fengycin A **(B)**, and surfactin **(C)**.

Quadrupole time-of-flight mass spectrometry analysis indicated five types of lipopeptides and two predominant compounds. This analysis detected a [M+H] peak at m/z 1022.6729 and afforded the molecular formula C_52_H_91_N_7_O_13_ (i-Fit = 20.6 and DBE = 10.5) and corresponding to surfactin and [M+H] peak at m/z 1463.8038 corresponding to fengycin A with the molecular formula C_72_H_110_N_12_O_20_ (i-Fit = 30.2 and DBE = 23.5) (**Figures 4C** and **4A**, respectively).

Quadrupole time-of-flight mass spectrometry analysis indicated five types of lipopeptides and two predominant compounds. This analysis detected a [M+H] peak at m/z 1031.5431 (1053.5276 corresponds to the complementary sodium adduct molecular ion [M+Na]) with the molecular formula C_48_H_75_N_10_O_15_ (i-Fit = 101.1 and DBE = 16.5) corresponding to bacillomycin D, a [M+H] peak at m/z 1463.8038 with the molecular formula C_72_H_110_N_12_O_20_ (i-Fit = 130.3 and DBE = 23.5) corresponding to fengycin A and a [M+H] peak at m/z 1022.6729 and afforded the molecular formula C_52_H_91_N_7_O_13_ (i-Fit = 161.5 and DBE = 10.5) and corresponding to surfactin (**Figures 4A**, **4B**, and **4C**, respectively).

### Effect of XT1 Lipopeptides on *B. cinerea* Mycelial Growth

**FIGURE 5:**
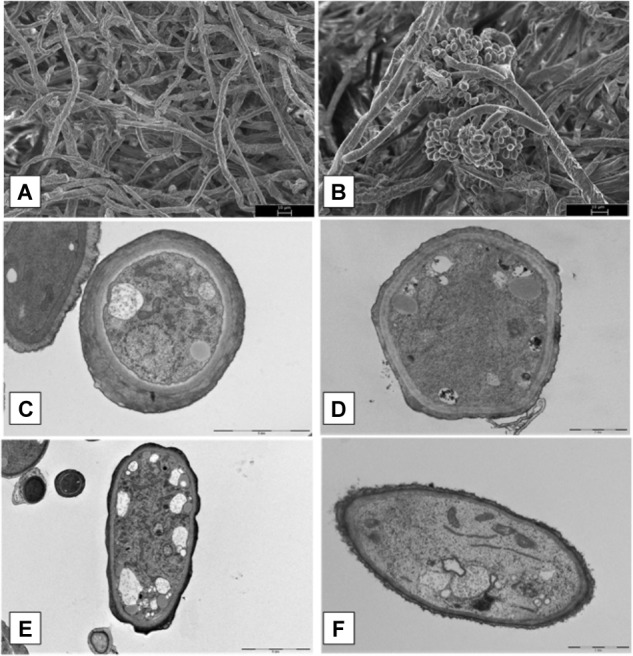
Scanning electron microscopy (SEM) and transmission electron microscopy (TEM) micrographs of hyphae of *Botrytis cinerea* treated with lipopeptides. Hyphae treated without and with lipopeptides (SEM) (**A,B**, respectively). Hyphae treated without and with lipopeptides (TEM) (**C,E** and **D,F**, respectively).

Microscopy data of *B. cinerea* mycelial growth treated with XT1 lipopeptides are shown in **Figure 5**. Scanning electron microscopy (SEM) analyses of *B. cinerea* mycelium treated with the MIC/MFC of lipopeptides (8 mg mL^-1^) from XT1 showed important alterations of pathogen morphology. Hyphae treated without lipopeptides grew normally with straight appearance and their surfaces were smooth (**Figure 5A**). However, after exposure to lipopeptides, one of the most striking features was the appearance of structures of resistance (**Figure 5B**).

Transmission electron microscopy (TEM) images of normal hyphae treated without lipopeptides showed smooth surfaces, intact cells, well defined enclosing cell walls and the cellular organelles in normal arrangements (**Figures 5C,E**). TEM images of hyphae confirmed that in the treatment of *B. cinerea* with lipopeptide organelles were degenerated and gathered in clumps (**Figures 5D,F**).

### Bioassay Against *B. cinerea* in Grapes, Strawberries, and Tomatoes

The MIC/MFC of lipopeptide extract from XT1 (8 mg mL^-1^) was applied in order to evaluate the protector effect with different fruit infected with *B. cinerea*. The lipopeptides were injected in grapes and sprayed onto strawberries and tomatoes. The level of disease infections decreased in all the fruit treated with XT1 lipopeptides.

**FIGURE 6:**
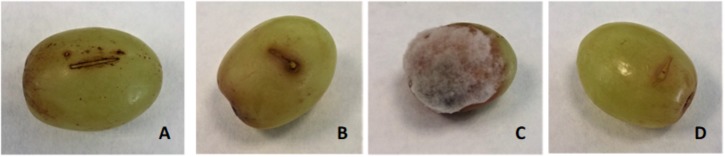
Lipopeptides from XT1 inhibited disease severity in grapes. Disease severity of grey mould on grapes. **(A)** Grapes treated with sterile water as the negative control. **(B)** Grapes treated with lipopeptides from XT1. **(C)** Grapes infected with *Botrytis cinerea*. **(D)** Grapes infected with *B. cinerea* and treated with lipopeptides from XT1.

**FIGURE 7:**
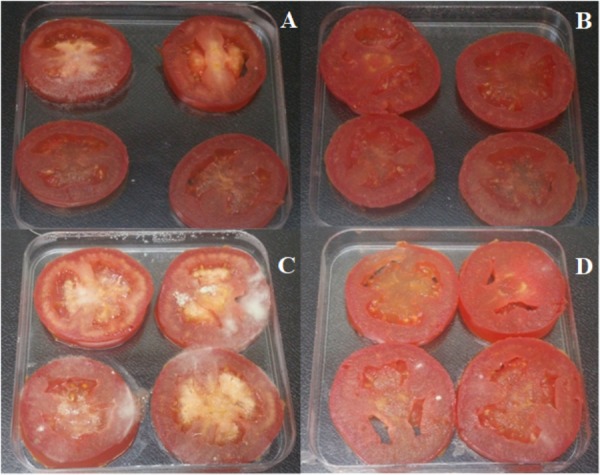
Lipopeptides from XT1 inhibited disease severity in tomatoes. Disease severity of grey mould on tomatoes. **(A)** Tomatoes treated with sterile water as the negative control. **(B)** Tomatoes treated with lipopeptides from XT1. **(C)** Tomatoes infected with *Botrytis cinerea*. **(D)** Tomatoes infected with *B. cinerea* and treated with lipopeptides from XT1.

The disease incidence in grapes, strawberries, and tomatoes treated with *B. cinerea* was 71, 100, and 100%, respectively. The disease reductions in fruit treated with XT1 lipopeptides were 100, 12, and 50%, respectively (**Table 3** and **Figures 6**, **7**). The results show that antifungal lipopeptide treated fruit reduced the disease symptoms significantly compared to non-treated fruits except in strawberries where the effect was not so evident. This reduction is higher for grapes.

**Table 3:** Disease incidence in grapes, strawberries, and tomatoes.

	Disease incidence (%)			
	
	Control	XT1 lipopeptides	*Botrytis*	XT1 lipopeptides + *Botrytis*
Grapes	0 ± 0.00a	0 ± 0.00a	71 ± 0.18b	0 ± 0.00a
Strawberry	43 ± 0.25b	33 ± 0.00a	100 ± 0.00d	88 ± 0.58c
Tomato	25 ± 0.20b	0 ± 0.17a	100 ± 0.00d	50 ± 0.13c


*Values followed by the same letters for each fruit did not differ significantly according to Duncan’s multiple range test (*p* < 0.05).*

### Antioxidant Activity on Fruit Treated With Lipopeptides

**FIGURE 8:**
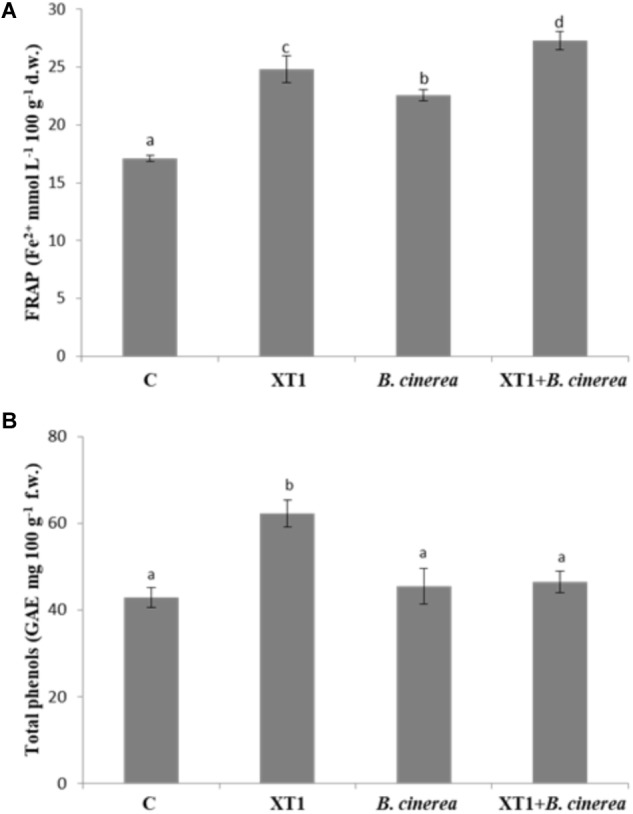
Antioxidant activity evaluated with the FRAP assay **(A)** and total phenols **(B)** of grapes treated with lipopeptides from XT1, infected with *Botrytis cinerea* and infected with *B. cinerea* but treated with lipopeptides from XT1. Values followed by the same letters did not differ significantly according to Duncan’s multiple range test (*p* < 0.05). Vertical lines represent the standard errors of the mean.

The antioxidant activity was tested in grapes where the highest disease reduction was observed. The antioxidant activity of grape extracts, as estimated by the FRAP assay increased significantly with the inoculation of the MIC/MFC of lipopeptides produced by XT1 (**Figure 8A**). Although an increase in the antioxidant activity was also observed in grapes infected with the pathogen, the highest increases were observed in the treatment with the lipopeptides.

On the other hand, total phenol content also increased with the exposure of fruit to the lipopeptides but the increase is only significant in the uninfected grapes. In this case the increase of total phenol content is of 30% (**Figure 8B**).

## Discussion

The strain *Bacillus* XT1 is a gram positive, sporulated, and halotolerant rod that grows in a wide range of salt concentrations (0–12% w/v), temperature (15–40°C), and pH (5–10) (Béjar et al., 2014). The strain was originally classified as *B. methylotrophicus* XT1 CECT 8661 (deposited according to the Bucharest Treaty for patenting purposes and licenced to Xtrem Biotech S.L.). The species *B. methylotrophicus* was later reclassified as a heterotypic synonym of *B. velezensis* (Dunlap et al., 2016).

This paper describes the antifungal activity of the strain and its lipopeptides against *B. cinerea*, a filamentous fungus classified as the second most important phytopathogen worldwide. Also demonstrate the implication of XT1 lipopeptides in the *in vivo* antibiosis and in the alteration of fungus structures and in the induced systemic response of fruits affected with the fungi.

One of the major factors related with the antifungal activity of members of the genus *Bacillus* is due to the production of lipopeptides synthesised by NRPSs such as iturin, fengycin, and surfactin (Romero et al., 2007; Ongena and Jacques, 2008; Pramudito et al., 2018). Lipopeptides are produced as a mixture of macromolecules belonging to the same family or class. Genomic analysis of *Bacillus* XT1 indicated that it contains gene clusters for non-ribosomal lipopeptide synthetases related to the production of surfactin, iturin, fengycin, and bacillomycin. Detection of the produced lipopeptides by XT1 was performed by Q-TOF MS analysis showing that strain XT1 produces all lipopeptides except iturin. Some of these macromolecules were previously shown to be produced by other *B. methylotrophicus* strains (Frikha-Gargouri et al., 2017).

Lipopeptides biosynthesis from XT1 was tested in different culture media. The data showed that the culture medium used in the growth of the microorganism could decisively influence the production of lipopeptides. Ahimou et al. (2000) described an optimum medium (named MOLP medium) for lipopeptide production by *Bacillus subtilis*. This study concluded that the medium MOLP is also the best to increase the lipopeptide production yields in *B. methylotrophicus* by *Bacillus* XT1. The influence of culture conditions on lipopeptide production was previously described for other strains of *Bacillus* genus like *B. subtilis* or *B. amyloliquefaciens* (Monteiro et al., 2005; Medeot et al., 2017). In terms of stability, the thermostable nature of XT1 lipopeptides and the fact that these antifungal compounds were affected by extremely alkaline pH were also observed in the evaluation of the activity of the *B. subtilis* biosurfactant (Ghribi et al., 2012).

Several studies have previously highlighted the antagonistic effect against different pathogens of NRP metabolites such as lipopeptides (Chowdhury et al., 2015; Mnif and Ghribi, 2015). For example, it has been demonstrated that the biocontrol of *Bacillus* strains against different pathogenic bacteria and fungi such as *Aspergillus* or *Pseudomonas syringae* is facilitated by lipopeptides such as surfactin (Bais et al., 2004). According to the literature, few studies describe the inhibitory activity of the lipopeptides produced by *Bacillus* strains against *B. cinerea.* All the studies reported the antibiosis of *B. cinerea* by the NRPs metabolites produced by *B. subtilis* but do not demonstrate directly the implications of these molecules in the antibiosis (Tareq et al., 2014; Farace et al., 2015; Wang et al., 2015; Arroyave-Toro et al., 2017). The same occurs with *B. amyloliquefaciens* (Ji et al., 2013; Pretorius et al., 2015; Tanaka et al., 2015; Zhang et al., 2017), *B. marinus* (Gu et al., 2017), *B. atrophaeus* (Zhang et al., 2013), and *B. velezensis* (Ge et al., 2016; Romero et al., 2016; Gao et al., 2017).

With respect to the activity of lipopeptides, Romano et al. (2013) reported the non-effect of *B. amyloliquefaciens* lipopeptides against *B. cinerea* at concentrations of 0.1 mg mL^-1^. Tareq et al. (2014) established a range of lipopeptide activity in *B. subtilis* between 0.5 to 300 μg mL^-1^ and Zhang et al. (2017) determined the maximum activity of lipopeptides of *B. amyloliquefaciens* at 30 mg mL^-1^. Our study tested a range of concentrations from 0.5 to 20 mg mL^-1^ and confirmed the antifungal activity of XT1 lipopeptides with inhibitory and fungicidal effects of these compounds at concentrations as low as 8 mg mL^-1^ (MIC and MFC). This fact and the higher productions of lipopeptides in MOLP medium may determine the high fungicidal activity of *Bacillus* XT1 against *B. cinerea.*

Different studies show that lipopeptides from *Bacillus* sp. strains produce damage to the hyphae and survival structures of pathogenic fungi (Souto et al., 2004). Chitarra et al. (2003) suggested that lipopeptides produced by *B. subtilis* YM 10-20 may permeabilize fungal spores and inhibit their germination. Other studies show the swelling and the deformation of fungus hyphae of *Pestalotiopsis eugeniae* when treated by the lipopeptides of *B. subtilis* BS-99-H (Lin et al., 2010). The effects of the lipopeptides produced by XT1 on the morphology of *B. cinerea* were evaluated in solid media. SEM studies revealed an extensive formation of fungal spores in the intersection of the fungus-bacteria inhibition zone. The same results were observed in previous studies where the biological activity of lipopeptides from *B. amyloliquefaciens* against *Fusarium solani* was analysed (Torres et al., 2017). Gong et al. (2014) studied the effect of bacillomycin D from *B. subtilis* on *Aspergillus flavus* and concluded that due to the amphipathic nature of bacillomycin D, this compound entered the spores and the hyphae where it caused pores to be formed in the membrane, resulting in the leakage of cell contents. TEM images of hyphae confirmed that in the treatment of *B. cinerea* with XT1 lipopeptides the organelles degenerated probably due to the entry of these compounds.

In this study, the antifungal activity of lipopeptides from XT1 against grey mould disease in different fruit was also investigated. The inoculation results showed that grey mould disease on grapes and tomatoes was significantly inhibited by the lipopeptides produced by XT1. Previous studies have described the involvement of lipopeptides from *B. subtilis* in grapevine plant defence and local resistance against *B. cinerea* (Farace et al., 2015). They also showed that lipopeptides are perceived by grapevine plant cells and activate different signalling pathways. Previous studies suggest that lipopeptides act as elicitors of defence-related genes (Waewthongrak et al., 2014). However, and to the best of our knowledge, our study is the first to highlight the ability of lipopeptides to trigger the antioxidant activity of these macromolecules in fruit. The total phenol content was increased significantly with the exposure of the fruit to the lipopeptides produced by XT1. The highest increases in antioxidant activity were observed in the infected fruits and in those treated with the lipopeptides. These data may suggest that the antimicrobial effect of lipopeptides and the accumulation of antioxidant compounds are closely related with pathogen resistance.

## Conclusion

In this study, we have investigated the high antifungal activity against *B. cinerea* of a patented strain, *Bacillus* XT1 CECT 8661. The lipopeptides produced by XT1 are involved in the biological control of *B. cinerea* and trigger the antioxidant activity in fruit. Based on the inhibitory effect on the development of grey mould on grapes and tomatoes, *Bacillus* XT1 CECT 8661 could be considered as a potential alternative for chemical fungicides in reducing the damage of grey mould disease.

## Author Contributions

LT carried out the experimental techniques and statistical analysis. MR collaborated in the experimental techniques related with determination of lipopeptide genes and analysis of the chromatograms. VB collaborated in the design of the techniques related with the extraction and the study of antifungal activity of lipopeptides, analysed the results, and critically read the manuscript. IS designed the experimental techniques, analysed the results, and drafted the manuscript.

## Conflict of Interest Statement

LT is a full-time employee of Xtrem Biotech S.L., company that holds an exclusive licence agreement on the patent that protects XT1 industrial exploitation. MR, VB, and IS declare that the research was conducted in the absence of any commercial or financial relationships that could be construed as a potential conflict of interest.
